# (1*RS*,2*RS*)-4,4′-(1-Aza­niumyl-2-hy­droxy­ethane-1,2-di­yl)dipyridinium tetra­chlorido­platinate(II) chloride

**DOI:** 10.1107/S160053681300425X

**Published:** 2013-02-20

**Authors:** José J. Campos-Gaxiola, Jorge L. Almaral-Sanchez, Adriana Cruz-Enríquez, Herbert Höpfl, Miguel Parra-Hake

**Affiliations:** a Facultad de Ingenieria Mochis, Universidad Autónoma de Sinaloa, Fuente Poseidón y Prol. A. Flores S/N, CP 81223, C.U. Los Mochis, Sinaloa, México; b Centro de Investigaciones Quimicas, Universidad Autónoma del Estado de Morelos, Av. Universidad 1001, CP 62210, Cuernavaca, Morelos, México; c Centro de Graduados e Investigación en Química del Instituto Tecnologico de Tijuana, Blvd. Industrial S/N, Col. Otay, CP 22500, Tijuana, B.C., México

## Abstract

The title compound, (C_12_H_16_N_3_O)[PtCl_4_]Cl, consists of a 4,4′-(1-aza­niumyl-2-hy­droxy­ethane-1,2-di­yl)dipyridinium trication, a square-planar tetra­chloridoplatinate(II) dianion and a chloride ion. In the cation, the pyridinium rings attached to the central 1-aza­niumyl-2-hy­droxy­ethane fragment have an *anti* conformation, as indicated by the central C—C—C—C torsion angle of −166.5 (6)°, and they are inclined to one another by 63.5 (4)°. In the crystal, the cations and anions are linked through N—H⋯Cl and O—H⋯Cl hydrogen bonds. There are also π–π contacts [centroid–centroid distances = 3.671 (4) and 3.851 (4) Å] and a number of C—H⋯Cl inter­actions present, consolidating the formation of a three-dimensional supra­molecular structure.

## Related literature

For potential applications of organic-inorganic hybrid materials with magnetic, optical and electrical properties, see: Yao *et al.* (2010 ▶); Sanchez *et al.* (2011 ▶); Pardo *et al.* (2011 ▶); Piecha *et al.* (2012 ▶). For related tetra­chloro­platinate(II) compounds, see: Fusi *et al.* (2012 ▶); Adarsh *et al.* (2010 ▶); Campos-Gaxiola *et al.* (2010 ▶); Adams *et al.* (2005 ▶). For the synthesis of the title ligand, see: Campos-Gaxiola *et al.* (2012 ▶).
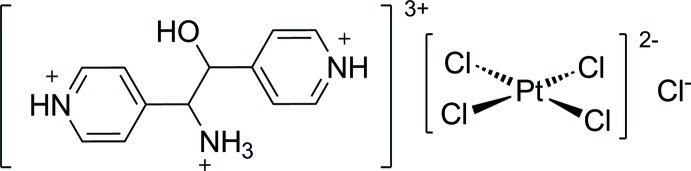


## Experimental

### 

#### Crystal data

(C_12_H_16_N_3_O)[PtCl_4_]Cl
*M* *_r_* = 590.62
Triclinic, 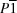

*a* = 7.636 (2) Å
*b* = 8.082 (2) Å
*c* = 14.599 (4) Å
α = 88.689 (4)°
β = 84.240 (4)°
γ = 70.148 (4)°
*V* = 843.1 (4) Å^3^
*Z* = 2
Mo *K*α radiation
μ = 9.12 mm^−1^
*T* = 100 K
0.50 × 0.26 × 0.12 mm

#### Data collection

Bruker SMART CCD area-detector diffractometer
Absorption correction: multi-scan (*SADABS*; Sheldrick, 1996 ▶) *T*_min_ = 0.092, *T*_max_ = 0.408
5093 measured reflections
2911 independent reflections
2726 reflections with *I* > 2σ(*I*)
*R*_int_ = 0.043

#### Refinement

*R*[*F* ^2^ > 2σ(*F* ^2^)] = 0.038
*wR*(*F* ^2^) = 0.091
*S* = 1.05
2911 reflections
217 parameters
6 restraints
H atoms treated by a mixture of independent and constrained refinement
Δρ_max_ = 2.34 e Å^−3^
Δρ_min_ = −1.98 e Å^−3^

### 

Data collection: *SMART* (Bruker, 2000 ▶); cell refinement: *SAINT-Plus-NT* (Bruker 2001 ▶); data reduction: *SAINT-Plus-NT*; program(s) used to solve structure: *SHELXS97* (Sheldrick, 2008 ▶); program(s) used to refine structure: *SHELXL97* (Sheldrick, 2008 ▶); molecular graphics: *ORTEP-3 for Windows* (Farrugia, 2012 ▶) and *Mercury* (Macrae *et al.*, 2008 ▶); software used to prepare material for publication: *publCIF* (Westrip, 2010 ▶).

## Supplementary Material

Crystal structure: contains datablock(s) I, global. DOI: 10.1107/S160053681300425X/su2560sup1.cif

Structure factors: contains datablock(s) I. DOI: 10.1107/S160053681300425X/su2560Isup2.hkl

Additional supplementary materials:  crystallographic information; 3D view; checkCIF report

## Figures and Tables

**Table 1:** Hydrogen-bond geometry (Å, °)

*D*—H⋯*A*	*D*—H	H⋯*A*	*D*⋯*A*	*D*—H⋯*A*
O1—H1′⋯Cl1^i^	0.84 (6)	2.49 (7)	3.250 (6)	152 (6)
N1—H1*A*⋯Cl5^ii^	0.86 (7)	2.32 (6)	3.148 (6)	162 (7)
N1—H1*B*⋯Cl5^iii^	0.86 (5)	2.30 (6)	3.097 (7)	154 (7)
N1—H1*C*⋯Cl2	0.86 (5)	2.50 (5)	3.214 (6)	141 (6)
N1—H1*C*⋯Cl3	0.86 (5)	2.58 (7)	3.242 (6)	134 (6)
N2—H2′⋯Cl5^iv^	0.84 (4)	2.45 (7)	3.088 (6)	134 (7)
N2—H2′⋯Cl5^v^	0.84 (4)	2.69 (6)	3.272 (6)	128 (7)
N3—H3′⋯Cl1^vi^	0.84 (6)	2.50 (6)	3.275 (6)	155 (6)
N3—H3′⋯Cl4^vi^	0.84 (6)	2.72 (7)	3.286 (7)	127 (7)
C1—H1⋯Cl1^vii^	0.98	2.71	3.660 (8)	163
C5—H5⋯Cl3^iii^	0.93	2.71	3.604 (8)	162
C10—H10⋯Cl3^i^	0.93	2.73	3.459 (8)	136
C10—H10⋯Cl5^v^	0.93	2.74	3.308 (7)	120
C11—H11⋯Cl2^viii^	0.93	2.64	3.449 (8)	145

Symmetry codes: (i) 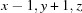; (ii) 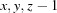; (iii) 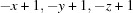; (iv) 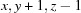; (v) 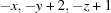; (vi) 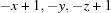; (vii) 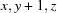; (viii) 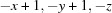.

**Fig. 1.**
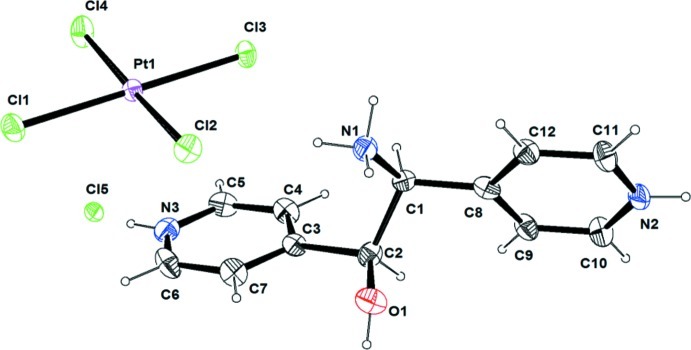
The molecular structure of the title compound, with atom labelling. Displacement ellipsoids are drawn at the 50% probability level.

**Fig. 2.**
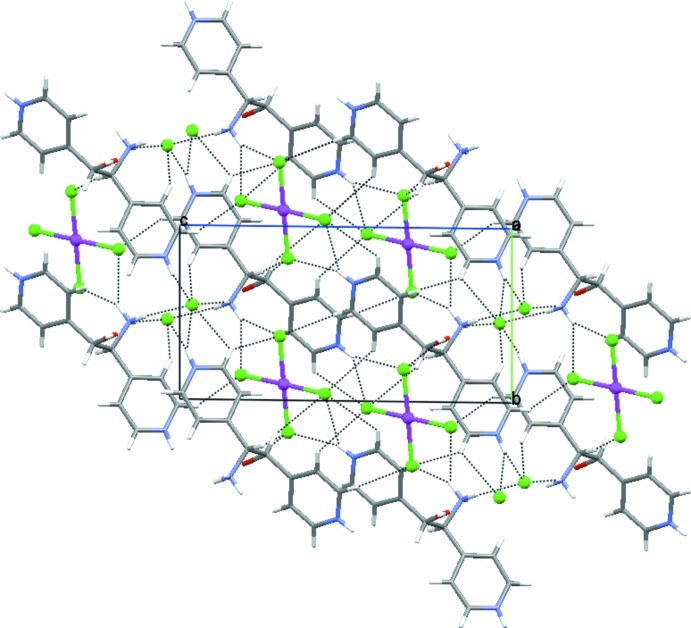
Perspective view of a fragment of the three-dimensional supramolecular network with O—H···Cl, N—H···Cl and C—H···Cl hydrogen bonds (dashed lines; see Table 1 for details).

## supplementary crystallographic information

### Comment

Hydrogen bond based organic–inorganic hybrid materials are receiving continuous interest because of their structural, magnetic, optical and electrical properties (Yao *et al.* 2010; Sanchez *et al.* 2011; Pardo *et al.* 2011 and Piecha *et al.* 2012). An interesting approach for the preparation of such materials consists in the utilization of supramolecular synthons containing charge-assisted N^+^–H···^-^Cl hydrogen bonds, through which organic cations and anionic metal complexes are linked to form crystalline organic–inorganic hybrid solids (Fusi *et al.* 2012; Adarsh *et al.* 2010; Campos-Gaxiola *et al.* 2010, and Adams *et al.* 2005). As a further contribution we report herein the crystal structure of the title compound.

The molecular structure of the title compound is illustrated in Fig. 1. The asymmetric unit consists of one threefold charged organic cation in a general position, one independent [PtCl_4_]^2-^ dianion, and one chloride atom (Fig 1). In the cation, the pyridinium rings attached to the central 2-ammoniumethanol fragment show *anti* conformation, as indicated by the C8—C1—C2—C3 torsion angle of -166.5 (6)°. The pyridinium rings form a dihedral angle of 63.5 (4)°. The Pt atom is embedded in a square-planar coordination environment with Pt—Cl distances ranging from 2.2999 (17) to 2.3127 (18) Å.

In the crystal, the cations and anions are linked by charge-assisted N^+^—H···^-^Cl, O—H···^-^Cl hydrogen bonds (Table 1). There are also a number of C—H···Cl contacts and π—π interactions present, consolidating the formation of the three-dimensional supramolecular structure (Table 1 and Fig 2). The π—π interactions are parallel slipped interactions involving inversion related pyridinium rings, Cg1 = N2/C8-C12 and Cg2 = N3/C3-C7 [Cg1···Cg1^i^ = 3.851 (4); normal distance 3.487 (3) Å; slippage 1.634 Å; symmetry code: (i) -x, -y+2, -z: Cg2···Cg2^ii^ = 3.671 (4) Å; normal distance 3.460 (3) Å; slippage 1.225 Å; symmetry code: (ii) -x, -y+1, -z].

### Experimental

The organic entities in the title compound are a product of partial hydrolysis starting from 2,4,5-tris(pyridin-4-yl)-4,5-dihydro-1,3-oxazole, which was synthesized according to a previously reported procedure (Campos-Gaxiola *et al.*, 2012). For the preparation of the platinum compound, a solution of 2,4,5-tris(pyridin-4-yl)-4,5-dihydro-1,3-oxazole (0.05 g, 0.16 mmol) in methanol and concentrated HCl (37%, 3 ml) was added dropwise to a stirring solution of potassium tetrachloroplatinate (0.06 g, 0.16 mmol) in water (5 ml). The resulting yellow solution was stirred for 40 Min at 323 K, whereupon the solution was left to evaporate slowly at room temperature. After two weeks, yellow crystals were isolated [Yield: 45%]. Spectroscopic and other analytical data for the title compound are available in the archived CIF.

### Refinement

The N—H and O—H hydrogen atoms were localized in difference Fourier maps. They were refined with distance restraints: O-H = 0.84 (1) Å, N-H = 0.86 (1) (NH_3_^+^) and 0.84 (1) Å (pyN-H^+^), with *U*_iso_(H) = 1.5 *U*_eq_(O, N). C-bound H atoms were positioned geometrically and refined using a riding-model approximation: aryl C—H = 0.93 Å, alkyl C—H = 0.98 Å with *U*_iso_(H) = 1.2 *U*_eq_(C).

### Crystal data

**Table:**

(C_12_H_16_N_3_O)[PtCl_4_]Cl	*Z* = 2
*M**_r_* = 590.62	*F*(000) = 560
Triclinic, *P*1	*D*_x_ = 2.326 Mg m^−^^3^
Hall symbol: -P 1	Mo *K*α radiation, λ = 0.71073 Å
*a* = 7.636 (2) Å	Cell parameters from 926 reflections
*b* = 8.082 (2) Å	θ = 2.7–27.5°
*c* = 14.599 (4) Å	µ = 9.12 mm^−^^1^
α = 88.689 (4)°	*T* = 100 K
β = 84.240 (4)°	Rectangular prism, orange
γ = 70.148 (4)°	0.50 × 0.26 × 0.12 mm
*V* = 843.1 (4) Å^3^	

### Data collection

**Table:**

Bruker SMART CCD area-detector diffractometer	2911 independent reflections
Radiation source: fine-focus sealed tube	2726 reflections with *I* > 2σ(*I*)
Graphite monochromator	*R*_int_ = 0.043
phi and ω scans	θ_max_ = 25.0°, θ_min_ = 2.7°
Absorption correction: multi-scan (*SADABS*; Sheldrick, 1996)	*h* = −8→9
*T*_min_ = 0.092, *T*_max_ = 0.408	*k* = −8→9
5093 measured reflections	*l* = −17→16

### Refinement

**Table:**

Refinement on *F*^2^	Primary atom site location: structure-invariant direct methods
Least-squares matrix: full	Secondary atom site location: difference Fourier map
*R*[*F*^2^ > 2σ(*F*^2^)] = 0.038	Hydrogen site location: inferred from neighbouring sites
*wR*(*F*^2^) = 0.091	H atoms treated by a mixture of independent and constrained refinement
*S* = 1.05	*w* = 1/[σ^2^(*F*_o_^2^) + (0.043*P*)^2^] where *P* = (*F*_o_^2^ + 2*F*_c_^2^)/3
2911 reflections	(Δ/σ)_max_ < 0.001
217 parameters	Δρ_max_ = 2.34 e Å^−^^3^
6 restraints	Δρ_min_ = −1.98 e Å^−^^3^

### Special details

**Table:**

Experimental. Spectroscopic and other analytical data for the title compound: IR (KBr, cm^-1^): 3409, 3198, 3071, 2882, 2825, 1706, 1620, 1500, 1417, 1331, 1295, 1232, 1031, 857 and 693. TGA: Calcd. for HCl: 4.32. Found: 4.75% (310–398 K); Calcd. for 2HCl: 8.65. Found: 8.23% (398–498 K).
Geometry. Bond distances, angles etc. have been calculated using the rounded fractional coordinates. All su's are estimated from the variances of the (full) variance-covariance matrix. The cell esds are taken into account in the estimation of distances, angles and torsion angles
Refinement. Refinement of *F*^2^ against ALL reflections. The weighted *R*-factor *wR* and goodness of fit *S* are based on *F*^2^, conventional *R*-factors *R* are based on *F*, with *F* set to zero for negative *F*^2^. The threshold expression of *F*^2^ > σ(*F*^2^) is used only for calculating *R*-factors(gt) *etc*. and is not relevant to the choice of reflections for refinement. *R*-factors based on *F*^2^ are statistically about twice as large as those based on *F*, and *R*- factors based on ALL data will be even larger.

### Fractional atomic coordinates and isotropic or equivalent isotropic displacement parameters (Å^2^)

**Table:**

	*x*	*y*	*z*	*U*_iso_*/*U*_eq_	
O1	−0.0077 (7)	0.6320 (7)	0.1932 (4)	0.0269 (17)	
N1	0.3686 (8)	0.5578 (8)	0.1506 (4)	0.0204 (17)	
N2	0.0968 (8)	1.1792 (8)	0.0449 (4)	0.0209 (19)	
N3	0.2400 (9)	0.3227 (9)	0.4748 (4)	0.025 (2)	
C1	0.2559 (9)	0.7175 (9)	0.2054 (5)	0.018 (2)	
C2	0.0855 (10)	0.6886 (9)	0.2597 (5)	0.021 (2)	
C3	0.1431 (9)	0.5553 (9)	0.3366 (5)	0.019 (2)	
C4	0.2175 (10)	0.5946 (10)	0.4125 (5)	0.023 (2)	
C5	0.2658 (10)	0.4784 (10)	0.4820 (5)	0.024 (2)	
C6	0.1694 (11)	0.2782 (10)	0.4047 (5)	0.027 (3)	
C7	0.1181 (10)	0.3947 (9)	0.3338 (5)	0.022 (2)	
C8	0.1965 (9)	0.8788 (9)	0.1453 (5)	0.019 (2)	
C9	0.0633 (10)	1.0334 (9)	0.1822 (5)	0.022 (2)	
C10	0.0133 (10)	1.1822 (9)	0.1300 (5)	0.022 (2)	
C11	0.2254 (10)	1.0358 (9)	0.0076 (5)	0.022 (2)	
C12	0.2759 (10)	0.8818 (9)	0.0559 (5)	0.020 (2)	
Pt1	0.63165 (3)	0.09264 (3)	0.31275 (2)	0.0146 (1)	
Cl1	0.6085 (3)	−0.1851 (2)	0.32558 (12)	0.0222 (6)	
Cl2	0.4705 (3)	0.1426 (2)	0.18394 (12)	0.0226 (5)	
Cl3	0.6558 (2)	0.3685 (2)	0.30103 (12)	0.0192 (5)	
Cl4	0.8053 (3)	0.0399 (2)	0.43690 (13)	0.0259 (6)	
Cl5	0.2607 (2)	0.4569 (2)	0.96236 (11)	0.0201 (5)	
H1	0.33450	0.73790	0.24990	0.0210*	
H1'	−0.117 (5)	0.650 (12)	0.218 (5)	0.0400*	
H1A	0.317 (10)	0.528 (10)	0.107 (4)	0.0310*	
H1B	0.480 (4)	0.560 (11)	0.137 (5)	0.0310*	
H1C	0.407 (11)	0.467 (6)	0.185 (4)	0.0310*	
H2	0.00200	0.80100	0.28680	0.0250*	
H2'	0.071 (11)	1.280 (4)	0.022 (5)	0.0320*	
H3'	0.273 (11)	0.258 (9)	0.520 (4)	0.0370*	
H4	0.23480	0.70270	0.41600	0.0270*	
H5	0.31520	0.50550	0.53290	0.0290*	
H6	0.15420	0.16900	0.40300	0.0320*	
H7	0.06670	0.36490	0.28420	0.0270*	
H9	0.00840	1.03550	0.24220	0.0260*	
H10	−0.07810	1.28470	0.15380	0.0270*	
H11	0.28160	1.03940	−0.05160	0.0270*	
H12	0.36310	0.78010	0.02890	0.0240*	

### Atomic displacement parameters (Å^2^)

**Table:**

	*U*^11^	*U*^22^	*U*^33^	*U*^12^	*U*^13^	*U*^23^
O1	0.026 (3)	0.035 (3)	0.023 (3)	−0.015 (3)	−0.001 (2)	−0.001 (2)
N1	0.020 (3)	0.021 (3)	0.018 (3)	−0.005 (3)	0.001 (3)	0.002 (3)
N2	0.022 (3)	0.013 (3)	0.027 (4)	−0.006 (3)	0.000 (3)	0.002 (3)
N3	0.020 (3)	0.029 (4)	0.020 (4)	−0.003 (3)	0.004 (3)	0.005 (3)
C1	0.015 (3)	0.019 (4)	0.018 (4)	−0.005 (3)	−0.002 (3)	0.000 (3)
C2	0.021 (4)	0.021 (4)	0.019 (4)	−0.004 (3)	−0.002 (3)	0.000 (3)
C3	0.018 (4)	0.024 (4)	0.011 (3)	−0.006 (3)	0.006 (3)	0.000 (3)
C4	0.027 (4)	0.022 (4)	0.019 (4)	−0.010 (3)	0.005 (3)	−0.007 (3)
C5	0.025 (4)	0.032 (4)	0.016 (4)	−0.012 (3)	0.001 (3)	−0.006 (3)
C6	0.036 (5)	0.023 (4)	0.023 (4)	−0.016 (4)	0.008 (3)	−0.003 (3)
C7	0.029 (4)	0.026 (4)	0.014 (4)	−0.012 (3)	−0.002 (3)	−0.004 (3)
C8	0.020 (4)	0.022 (4)	0.017 (4)	−0.011 (3)	0.001 (3)	−0.005 (3)
C9	0.026 (4)	0.020 (4)	0.020 (4)	−0.010 (3)	0.004 (3)	0.000 (3)
C10	0.030 (4)	0.018 (4)	0.017 (4)	−0.007 (3)	0.004 (3)	−0.003 (3)
C11	0.032 (4)	0.022 (4)	0.016 (4)	−0.013 (3)	−0.002 (3)	0.002 (3)
C12	0.021 (4)	0.020 (4)	0.018 (4)	−0.007 (3)	0.003 (3)	−0.003 (3)
Pt1	0.0164 (2)	0.0135 (2)	0.0136 (2)	−0.0049 (1)	−0.0005 (1)	−0.0017 (1)
Cl1	0.0271 (10)	0.0188 (9)	0.0244 (10)	−0.0119 (8)	−0.0050 (7)	0.0010 (7)
Cl2	0.0248 (10)	0.0238 (9)	0.0193 (9)	−0.0077 (8)	−0.0047 (7)	−0.0010 (7)
Cl3	0.0255 (9)	0.0136 (8)	0.0182 (9)	−0.0068 (7)	0.0000 (7)	−0.0018 (6)
Cl4	0.0365 (11)	0.0214 (9)	0.0243 (10)	−0.0127 (8)	−0.0144 (8)	0.0043 (7)
Cl5	0.0198 (9)	0.0204 (9)	0.0201 (9)	−0.0076 (7)	0.0006 (7)	0.0024 (7)

### Geometric parameters (Å, º)

**Table:**

Pt1—Cl2	2.303 (2)	C3—C4	1.384 (11)
Pt1—Cl3	2.2999 (17)	C3—C7	1.377 (10)
Pt1—Cl1	2.3127 (18)	C4—C5	1.358 (11)
Pt1—Cl4	2.300 (2)	C6—C7	1.378 (10)
O1—C2	1.428 (10)	C8—C12	1.386 (10)
O1—H1'	0.84 (6)	C8—C9	1.394 (10)
N1—C1	1.484 (9)	C9—C10	1.370 (10)
N2—C11	1.324 (9)	C11—C12	1.372 (10)
N2—C10	1.335 (9)	C1—H1	0.9800
N3—C6	1.314 (10)	C2—H2	0.9800
N3—C5	1.346 (10)	C4—H4	0.9300
N1—H1B	0.86 (5)	C5—H5	0.9300
N1—H1C	0.86 (5)	C6—H6	0.9300
N1—H1A	0.86 (7)	C7—H7	0.9300
N2—H2'	0.84 (4)	C9—H9	0.9300
N3—H3'	0.84 (6)	C10—H10	0.9300
C1—C2	1.539 (11)	C11—H11	0.9300
C1—C8	1.517 (10)	C12—H12	0.9300
C2—C3	1.529 (10)		
			
Cl3—Pt1—Cl4	89.28 (6)	C3—C7—C6	119.8 (7)
Cl1—Pt1—Cl4	90.27 (7)	C9—C8—C12	118.2 (6)
Cl1—Pt1—Cl2	90.20 (6)	C1—C8—C9	119.2 (6)
Cl1—Pt1—Cl3	179.55 (7)	C1—C8—C12	122.6 (6)
Cl2—Pt1—Cl3	90.24 (6)	C8—C9—C10	119.9 (7)
Cl2—Pt1—Cl4	177.33 (8)	N2—C10—C9	119.6 (7)
C2—O1—H1'	105 (5)	N2—C11—C12	120.3 (7)
C10—N2—C11	122.4 (6)	C8—C12—C11	119.6 (7)
C5—N3—C6	123.5 (7)	C8—C1—H1	108.00
C1—N1—H1C	112 (4)	N1—C1—H1	108.00
H1A—N1—H1B	117 (7)	C2—C1—H1	108.00
C1—N1—H1A	117 (5)	C3—C2—H2	109.00
H1B—N1—H1C	93 (8)	O1—C2—H2	109.00
C1—N1—H1B	109 (5)	C1—C2—H2	109.00
H1A—N1—H1C	107 (7)	C3—C4—H4	119.00
C11—N2—H2'	125 (5)	C5—C4—H4	119.00
C10—N2—H2'	112 (5)	N3—C5—H5	121.00
C6—N3—H3'	124 (5)	C4—C5—H5	121.00
C5—N3—H3'	113 (5)	C7—C6—H6	120.00
N1—C1—C8	111.6 (6)	N3—C6—H6	120.00
N1—C1—C2	110.4 (6)	C3—C7—H7	120.00
C2—C1—C8	111.2 (6)	C6—C7—H7	120.00
O1—C2—C1	105.6 (6)	C8—C9—H9	120.00
O1—C2—C3	112.3 (6)	C10—C9—H9	120.00
C1—C2—C3	111.8 (6)	C9—C10—H10	120.00
C2—C3—C7	121.2 (7)	N2—C10—H10	120.00
C2—C3—C4	120.9 (6)	N2—C11—H11	120.00
C4—C3—C7	118.0 (7)	C12—C11—H11	120.00
C3—C4—C5	121.3 (7)	C11—C12—H12	120.00
N3—C5—C4	118.0 (7)	C8—C12—H12	120.00
N3—C6—C7	119.5 (7)		
			
C11—N2—C10—C9	1.2 (12)	C1—C2—C3—C4	68.4 (9)
C10—N2—C11—C12	1.0 (12)	C1—C2—C3—C7	−113.4 (8)
C6—N3—C5—C4	−0.6 (12)	C2—C3—C4—C5	178.7 (7)
C5—N3—C6—C7	0.1 (12)	C7—C3—C4—C5	0.4 (12)
N1—C1—C2—O1	−53.4 (7)	C2—C3—C7—C6	−179.2 (7)
N1—C1—C2—C3	69.1 (7)	C4—C3—C7—C6	−0.9 (11)
C8—C1—C2—O1	71.1 (7)	C3—C4—C5—N3	0.3 (12)
C8—C1—C2—C3	−166.5 (6)	N3—C6—C7—C3	0.7 (12)
N1—C1—C8—C9	168.8 (7)	C1—C8—C9—C10	177.5 (7)
N1—C1—C8—C12	−14.3 (10)	C12—C8—C9—C10	0.5 (11)
C2—C1—C8—C9	45.1 (9)	C1—C8—C12—C11	−175.3 (7)
C2—C1—C8—C12	−138.1 (7)	C9—C8—C12—C11	1.6 (11)
O1—C2—C3—C4	−173.2 (7)	C8—C9—C10—N2	−1.9 (12)
O1—C2—C3—C7	5.1 (10)	N2—C11—C12—C8	−2.4 (12)

### Hydrogen-bond geometry (Å, º)

**Table:**

*D*—H···*A*	*D*—H	H···*A*	*D*···*A*	*D*—H···*A*
O1—H1′···Cl1^i^	0.84 (6)	2.49 (7)	3.250 (6)	152 (6)
N1—H1*A*···Cl5^ii^	0.86 (7)	2.32 (6)	3.148 (6)	162 (7)
N1—H1*B*···Cl5^iii^	0.86 (5)	2.30 (6)	3.097 (7)	154 (7)
N1—H1*C*···Cl2	0.86 (5)	2.50 (5)	3.214 (6)	141 (6)
N1—H1*C*···Cl3	0.86 (5)	2.58 (7)	3.242 (6)	134 (6)
N2—H2′···Cl5^iv^	0.84 (4)	2.45 (7)	3.088 (6)	134 (7)
N2—H2′···Cl5^v^	0.84 (4)	2.69 (6)	3.272 (6)	128 (7)
N3—H3′···Cl1^vi^	0.84 (6)	2.50 (6)	3.275 (6)	155 (6)
N3—H3′···Cl4^vi^	0.84 (6)	2.72 (7)	3.286 (7)	127 (7)
C1—H1···Cl1^vii^	0.98	2.71	3.660 (8)	163
C5—H5···Cl3^iii^	0.93	2.71	3.604 (8)	162
C10—H10···Cl3^i^	0.93	2.73	3.459 (8)	136
C10—H10···Cl5^v^	0.93	2.74	3.308 (7)	120
C11—H11···Cl2^viii^	0.93	2.64	3.449 (8)	145

Symmetry codes: (i) *x*−1, *y*+1, *z*; (ii) *x*, *y*, *z*−1; (iii) −*x*+1, −*y*+1, −*z*+1; (iv) *x*, *y*+1, *z*−1; (v) −*x*, −*y*+2, −*z*+1; (vi) −*x*+1, −*y*, −*z*+1; (vii) *x*, *y*+1, *z*; (viii) −*x*+1, −*y*+1, −*z*.
